# Social and Physical Environmental Factors Influencing Adolescents’ Physical Activity in Urban Public Open Spaces: A Qualitative Study Using Walk-Along Interviews

**DOI:** 10.1371/journal.pone.0155686

**Published:** 2016-05-23

**Authors:** Linde Van Hecke, Benedicte Deforche, Delfien Van Dyck, Ilse De Bourdeaudhuij, Jenny Veitch, Jelle Van Cauwenberg

**Affiliations:** 1 Department of Public Health, Faculty of Medicine and Health Sciences, Ghent University, Ghent, Belgium; 2 Physical activity, nutrition and health research unit, Department of Movement and Sport Sciences, Faculty of Physical Education and Physical Therapy, Vrije Universiteit Brussel, Brussels, Belgium; 3 Fund for Scientific Research Flanders (FWO), Brussels, Belgium; 4 Department of Movement and Sports Sciences, Faculty of Medicine and Health Sciences, Ghent University, Ghent, Belgium; 5 Institute for Physical Activity and Nutrition (IPAN), Deakin University, Geelong, Australia; Institute for Health & the Environment, UNITED STATES

## Abstract

Most previous studies examining physical activity in Public Open Spaces (POS) focused solely on the physical environment. However, according to socio-ecological models the social environment is important as well. The aim of this study was to determine which social and physical environmental factors affect adolescents’ visitation and physical activity in POS in low-income neighbourhoods. Since current knowledge on this topic is limited, especially in Europe, qualitative walk-along interviews were used to obtain detailed and context-specific information. Participants (n = 30, aged 12–16 years, 64% boys) were recruited in POS in low-income neighbourhoods in Brussels, Ghent and Antwerp (Belgium). Participants were interviewed while walking in the POS with the interviewer. Using this method, the interviewer could observe and ask questions while the participant was actually experiencing the environment. All audio-recorded interviews were transcribed and analysed using Nvivo 10 software and thematic analysis was used to derive categories and subcategories using a grounded theory approach. The most important subcategories that were supportive of visiting POS and performing physical activity in POS were; accessibility by foot/bicycle/public transport, located close to home/school, presence of (active) friends and family, cleanliness of the POS and features, availability of sport and play facilities, large open spaces and beautiful sceneries. The most important subcategories that were unsupportive of visiting POS and physical activity in POS were; presence of undesirable users (drug users, gangs and homeless people), the behaviour of other users and the cleanliness of the POS and features. Social factors appeared often more influential than physical factors, however, it was the combination of social and physical factors that affected adolescents’ behaviour in POS. Easily accessible POS with high quality features in the proximity of adolescents’ home or school may stimulate physical activity, if adolescents also experience a safe and familiar social environment.

## Introduction

Regular physical activity has well-documented physical, mental and social benefits on adolescent health [1, 2]. Despite these well-known benefits there has been a decline during the past decades in physical activity levels of adolescents (12–18 years) worldwide [3–5] and in Flanders (Belgium) [6, 7]. The low levels of physical activity reported in adolescents [3, 4] are even lower in adolescents with low socio-economic status (SES) [6–10]. Adolescents with low SES have reduced opportunities to be active with fewer recreational resources near home [11] and are less likely to be a member of a sports club [6, 9, 12]. Moreover, adolescents with low SES experience more barriers to be active such as; high cost of activities, high safety concerns, less parental support and more family responsibilities (e.g., looking after younger siblings) [13, 14].

According to ecological models of health behaviour, physical activity is influenced by individual, social and physical environmental characteristics [15]. The physical and social environment where adolescents are active differs from that of children and adults. Adolescents aged less than 16 years are often restricted to the nearby neighbourhood due to their limited independent mobility (restriction to visit a POS located far from home unaccompanied by an adult and in Belgium the minimum age for driving a motorbike is 16 years) [16, 17]. However, adolescents have more independence compared with younger children [16] and they tend to spend a lot of their time outdoors with friends and family [18]. Considering these specificities of adolescents, public open spaces (POS) may be a promising setting to promote adolescents’ physical activity [19, 20].

A POS is a public space with open access, that is accessible to all people independent of age, ethnicity, physical limitations, or other characteristics [21–23]. Some POS are under public ownership and management whereas others are private property but freely accessible and, therefore, also defined as a POS [23, 24]. POS can have different appearances such as parks, playgrounds and squares, but also streets, vacant lots and parking lots. In previous years, research on the relationship between the physical environment and physical activity in adolescence has increased [25–28], with studies reporting contradictory results on the importance of the accessibility and availability of parks, recreational facilities, opportunities to exercise and physical activity facilities [25, 28–32]. Some found no association [25, 28, 32, 33], whereas others found a positive association between proximity to parks, availability of recreational facilities, opportunities to be active and physical activity among youth [29–31]. These inconsistent results may indicate that other POS characteristics such as specific physical features of POS (e.g., trees that provide shade), quality of features, and the social environment (e.g., use by friends) play an important role in determining whether or not adolescents visit and use a POS for physical activity.

Little is known about the specific characteristics of POS that influence physical activity in adolescents. A previous study showed positive associations for the presence of trees providing shade and presence of signs regarding dogs with adolescent girls’ leisure-time physical activity in POS [20]. Most other research focused on one specific location such as parks or green space [34–36]. Furthermore, little is known about the social aspects of POS that are related to adolescents’ physical activity. Some research has focused on the social aspects of parks; park use by friends was associated with park use among adolescents [35] and opportunities for social interactions made parks more attractive for adolescent girls [34].

Besides physical activity in POS, active transportation (walking and cycling) could also contribute to overall physical activity levels. Most adolescents’ trips to parks are performed by foot or bike [17], and adolescents’ active transportation is positively associated with daily physical activity [37]. Accordingly, POS that are attractive to visit may increase overall physical activity levels (physical activity in the POS and active transportation to the POS).

Some previous studies included both factors of the physical and social environment [18, 36, 38–40]. However, the interplay between the social and physical environment remains unclear [18, 19, 35, 41, 42]. This may be due to the fact that these studies only included a few social environmental factors (e.g., crime or park use by friends) and these factors were not context-specific (i.e., they were not measured in the POS that were actually used). Limited research has studied social and physical environmental characteristics of POS simultaneously and this has not been studied yet in Europe. This indicates that there is need for more research on the physical and social characteristics of POS related to physical activity in adolescents.

Most research conducted in this area is quantitative survey research [20, 25, 27, 43]. However, the importance of physical aspects of POS are difficult to measure quantitatively because they are subject to various social factors and vice-versa. Qualitative research can provide more in-depth information and the opportunity to gain insight in to which characteristics of POS influence visitation, and how these characteristics may influence physical activity [15, 44]. Individual or focus group interviews are often used for qualitative research [38]. However, this method requires that the interviewee remembers all the impressions, experiences and events that happened in the POS while he/she is not present in the POS. Consequently, it is difficult to obtain in-depth information on the physical and social environmental context and information may be lost. Conducting interviews while walking within the POS (a walk-along interview), means that it is possible to study participants’ interpretation of the POS while experiencing it and thus uses the advantages of both face-to-face interviews and observations [44]. This methodology was previously utilized in a younger age group (7-to-9-year-olds) by Loebach and colleagues, to capture children’s use and perceptions of their neighbourhood [45]. This methodology creates an interplay between the environment (both social and physical), the researcher and the participant which can lead to in-depth information and new insights in the interplay between these environments.

This study used walk-along interviews in POS located in low SES neighbourhoods. The aim of this study was to identify the physical and social environmental factors influencing adolescents’ visitation and physical activity in POS located in neighbourhoods with low SES.

## Methods

### Participants and setting

Adolescents aged 12 to 16 years were recruited by face-to-face contact in POS in low SES neighbourhoods through purposeful convenience sampling until saturation of information was reached (final sample size = 30). When the participants agreed to participate, the interview was immediately conducted at the POS where they were recruited. Participants were recruited from thirteen different POS (3 squares, 2 skate parks, 6 parks and 2 sport fields/playgrounds) in eight neighbourhoods. Streets, vacant lots and parking lots were also visited to recruit participants. However, at these POS no adolescents were found. The POS were selected based on the advice of a youth worker and were located in three different Flemish cities (Brussels, Antwerp and Ghent) which are located within a radius of 50 km from each other. The size of the POS ranged from 507m² (square) to 190,760m² (park) with a mean area of 42,347m². Data collection was performed during daytime, on weekdays after school and on weekend days from July to October 2014 in neutral to good (i.e., not raining) weather conditions.

Low SES neighbourhoods/communities were selected based on population density, unemployment rates, welfare index and per capita income [46]. Population density (ranging from 6,920 inh/km² to 26,193 inh/km²) and unemployment rates (11%-36%) of the selected neighbourhoods were higher than average city values. Per capita income (€10,767-€15,913) was lower than average city values. The welfare-index ranged from 62–103. This is an indication of the income of the neighbourhood/community compared to the mean income in Belgium, with 100 considered a good welfare. The percentage of people with a nationality other than Belgian residing in the neighbourhood ranged from 22% to 46% [47–50].

Because 12 to 16 year olds were interviewed on a non-sensitive topic, it was opted to obtain an active consent from the participants and a passive consent from the parents or guardians [51, 52]. Participants indicated their consent by signing an informed consent form after the informed consent was read to them (to make sure all participants fully understood the written consent), additionally verbal consent was also given and audiotaped. The parents or guardians were given the opportunity to refuse participation of their children through a letter that was given to the participants. Without refusal, consent was assumed. This consent procedure and the research protocol for minors were approved by the medical ethics committee of the University Hospital of the Vrije Universiteit Brussel (BUN 143201420501) and the medical ethics committee of the University Hospital of Ghent University (EC/068-2015/mf) referring to the privacy act of December 8th, 2012 on the protection of privacy in relation to the processing of personal data [53]. Informed consent was obtained from all participants and all participants gave permission to use their quotes in research publications.

### Procedure and measurements

The interview consisted of three consecutive parts which were all audiotaped. The first component of the interview included questions that assessed demographics (age, sex, place of birth), frequency of visiting a POS (in a usual week), duration of a usual visit, activities in POS, satisfaction with number of POS in the neighbourhood, transport modes to POS, sport club membership and frequency of attending sport club and SES (parents’ jobs [54]). Participants were defined as low SES when none of the parents performed a white collar job.

Second, questions assessing the social environment were asked. These open-ended questions are described in Table 1 and were used to prompt a conversation about the social environment and to gain more insight and in-depth information on social context, modelling, social networks and social trust and cohesion.

**Table 1:** Questions used to assess the social context.

Topic	Questions
Social context	With whom do you come here?, Are there gangs hanging around?, Is there a lot of drug use?
Modelling [55, 56]	Are your friends and family active?, Do your friends often ask to hang out?, Are there lots of other people active here?
Social network [55, 57]	Do you know lots of people in the neighbourhood?, Are there lots of other adolescents to do things with?
Social trust and cohesion [56, 58]	Are people around here willing to help their neighbours?, Is it a close-knit neighbourhood?, Can people in this neighbourhood be trusted?, Do people in your neighbourhood generally get along well?

Third, a semi-structured walk-along interview was conducted in the POS where participants were recruited. The participant and researcher walked through the POS while conducting the walk-along interview. These walk-along interviews were conducted in Dutch and lasted 30–40 minutes.

Before starting the walk, the following instructions were read: *“We are now on place X*. *We would like to know more about the characteristics of this POS that encourage or discourage you to visit this POS and about the characteristics that encourage or discourage you to be active at this POS*. *First*, *you can tell us about the characteristics that are encouraging or discouraging to visit*, *and secondly you can tell us about characteristics that are encouraging or discouraging to be active*. *By physically active we mean all but sitting activities*. *This includes for example standing*, *playing games*, *doing sports*, *recreational activities or exercise*. *Think about the things that are more or less pleasant*, *interesting or attractive to visit or to be active on this space*. *What makes it fun to be here and to be active here? Also consider things that affect your feelings of safety*. *This can include safety from traffic and safety from crime*, *but also safety of being injured*. *Thus*, *think about all positive and negative things in this place that affect how you experience visiting and being active*. *You are the expert and it is the purpose that you tell us freely about your experiences*, *ideas and opinions*, *so that we can learn about the things in this POS that are encouraging or discouraging for you to visit or to be active here*. *Therefore*, *we may ask some additional questions to completely understand your experiences*, *ideas and opinions*. *All the information gathered will be strictly confidential and will only be used for our research*. *All things that you talk about*, *will be photographed*. *Is everything clear to you? As it is too difficult to write down the complete interview*, *it will be tape recorded*. *Do you agree with this*?*”*

When participants did not have the tendency to share experiences and opinions spontaneously, the interviewer stopped at regular (and random) moments during the walk and asked the following questions: *Are there characteristics that are encouraging or discouraging to visit*, *or to be active? Think about the things that are more or less pleasant*, *interesting or attractive to visit or to be active in this space*.

Participants who completed the walk-along interview were also asked if they were willing to complete a second interview at a POS that they do not usually visit to capture reasons for not visiting that POS. This second POS was selected by the interviewer after consulting with the participant and this second interview was conducted directly after the first interview. Seven participants were willing to participate in this second walk-along interview.

### Data analysis

Data from the first and second part of the interview were used to calculate descriptive statistics in SPSS version 22. Qualitative data from the audiotaped walk-along interviews and the social environmental questions were transcribed verbatim and analysed using Nvivo 10 software. Data analysis was guided by a grounded theory approach, which consists of systematic, yet flexible, guidelines for collecting and analysing qualitative data to construct theories from the data [59]. First, the walk-along interviews were read carefully, followed by inductive coding and assigning all mentioned physical and social environmental factors to subcategories. These subcategories were identified during the transcribing process based on frequently recurring themes. Finally, these subcategories were grouped into more general categories (Table 2). The assignment of the subcategories and grouping into categories was performed by two researchers (LVH and JVC) and disagreements were discussed until agreement was reached. For the physical environment, the categories were named consistent with previous literature on characteristics of parks associated with park use and physical activity [60, 61]. These categories include accessibility and location, features, aesthetics, upkeep, safety and policy.

**Table 2:** Overview of the physical and social environmental factors affecting visitation and physical activity in POS.

PHYSICAL ENVIRONMENT		Mentioned by few/some/many/almost all participants			
Categories	Subcategories	few	some	many	almost all
Accessibility and location	Close to home			X	
	Close to other locations		X		
	Presence of pubs and restaurants^a^	X			
	Well-known location	X			
	Accessibility by foot, bike and public transport				X
Features	Natural features				X
	Man-made facilities				X
	Diverse facilities for all ages		X		
Aesthetics	Beautiful scenery (nature and green)			X	
	Colour and graffiti^a^	X			
	Historical Elements	X			
	Noise^a^/quietness	X			
Upkeep	Cleanliness			X	
	Upkeep of facilities and playing surfaces		X		
Physical aspects of safety	Lighting	X			
	Safety from being hurt (maintenance of facilities)		X		
	Safety from traffic		X		
	Secluded areas^a^	X			
	Safety of accommodation for young children	X			
Policy	Organized activities		X		
	Secluded area for dogs	X			
**SOCIAL ENVIRONMENT**					
Categories	Subcategories	few	some	many	almost all
Social network	Friends and family				X
	Nice atmosphere	X			
	Knowing lots of people in the neighbourhood				X
	Other adolescents to play with/Social contact				X
Other Users	Behaviour of other users^a^				X
	Ethnicity of other users	X			
	Number of other users		X		
Social aspects of safety	Undesirable users^a^				X
	Presence of other people	X			
	Safety at night		X		
Parents	Habit (taught by parents)	X			
	Rules from parents		X		
Privacy	Privacy		X		
Modelling	Active use by others			X	
	Family active	X			
	Friends active				X

A few = topic mentioned by < 25% of participants, Some = topic mentioned by 25%-50% of participants, Many = topic mentioned by 50%-75% of participants, Almost all = topic mentioned by >75% of participants.

All subcategories mentioned in the table were mentioned by the participants to encourage POS visitation and/or physical activity unless stated otherwise.

^a^ Categories that were mostly mentioned to discourage POS visitation and/or physical activity.

Qualitative research can be supported by using quantitative counts of the number of times a certain topic was mentioned to indicate certain patterns or emphasize recurring themes. As recommended by Sandelowski (2001), the following classifications for cited topics were used: when a topic was mentioned by less than 25% of the participants it was referred to in the text as “a few”, by 25%-50% it was referred to as “some”, by 50%-75% it was referred to as “many” and by >75% it was referred to as “almost all” [62]. This approach has been used previously in research with similar methodologies by Van Cauwenberg et al. [63].

## Results

### Descriptives

Descriptive characteristics of the sample are presented in Table 3. The sample (n = 30), aged 13.3 ± 1.1 years, consisted of 63.3% boys and 86.7% of the participants were born in Belgium. All participants lived in an urban (>600inh./km²) or suburban (300-600inh./km²) [64] area and 62.5% of participants had a low SES based on occupation of both parents. Almost all participants (86.7%) visited a POS at least once a week and stayed there almost 2.5 hours. The most popular activities in POS (boys and girls) were soccer and basketball. An overview of the activities that participants engaged in, is presented in Table 4. Many participants (66.7%) were satisfied with the number of POS in the neighbourhood. Transport modes used to travel to a POS were walking (82.8%), bike/skateboard/rollerblade (31.0%) or public transportation (24.1%). Only 10.3% of the participants indicated that they travelled to a POS by car. In this sample, 46.7% of the participants were members of a sport club and attended this club on average 3.6 times per week.

**Table 3:** Descriptive statistics of the sample (n = 30).

**Demographics**	
Age (years) (M ± SD)	13.3 ± 1.1
Girls (%)	36.7
Born in Belgium (%)	86.7
Occupation Father (%)	
Blue collar worker	45.8
White collar worker	29.2
No Principal occupation	25.0
Occupation Mother (%)	
Blue collar worker	38.5
White collar worker	11.5
No Principal occupation	50.0
**Physical activity and POS use**	
POS visitation at least once a week (%)	86.7
Average duration of stay on a usual visit at the POS (M ± SD)	146.8 ± 90.2
Satisfied with the number of POS in neighbourhood (%)	66.7
Mode of transport to travel to a POS (%)	
Walking	82.8
Bike/skateboard/roller-skates	31.0
Public transport	24.1
Car	10.3
Active member of a sport club (%)	46.7
Frequency attending sports club (times/week) (M ± SD)	3.6 ± 3.2
**Social context**	
Accompaniment to POS (multiple answers possible) (%)	
Alone	14.3
Friends	71.4
Family	46.4
Organization	3.6
Participants indicating drug use in the POS (%)	63.3
Participants indicating gangs hanging around in POS (%)	60.0

**Table 4:** Activities performed in POS by the participants.

Girls (n = 11)	%(n)	Boys (n = 19)	%(n)
Dancing	9.1 (1)	Baseball	5.3 (1)
Frisbee	9.1 (1)	Climbing trees	5.3 (1)
Playground	9.1 (1)	Fantasy games	5.3 (1)
Roller-skating	9.1 (1)	Tennis^a^	5.3 (1)
Sledging (winter)	9.1 (1)	Badminton	10.5 (2)
Sitting	9.1 (1)	Playground	10.5 (2)
Tag	9.1 (1)	Hide and seek	15.8 (3)
Talking with family	9.1 (1)	Skateboarding	15.8 (3)
Ball games	18.2 (2)	Tag	15.8 (3)
Basketball	18.2 (2)	Jogging	21.1 (4)
Fantasy games	18.2 (2)	Table tennis	21.1 (4)
Go for a walk	18.2 (2)	Basketball	31.6 (6)
Jogging	18.2 (2)	Soccer	78.9 (15)
Soccer	27.3 (3)		

Participants were asked what kind of activities they engaged in when they went to the POS where the interview took place. They could provide as many answers as they preferred.

^a^ Tennis court was not available, therefore, they played tennis on the grass field.

### Part 1: Physical environment

#### Accessibility and location

Almost all participants mentioned at least one aspect of accessibility of the POS where they were interviewed. POS located close to their home were visited more often than POS located further away. Almost all participants indicated that good access by foot, bike and public transportation was important for visiting a POS. Some participants indicated that POS located close to other destinations such as schools, shops, the city centre or a friend’s house were used more frequently because they like to combine multiple activities. Additionally, well-known centrally located POS were easier to meet up with other friends. For example, one 15-year-old girl said: *“This is the park closest to my home and a lot of my friends live nearby*. *This is the place where we meet up because it is close for everyone*. *… Sometimes we go to those shops over there or we take the bus from here*.*”* (Girl 1, 15 years)

The presence of shops nearby a POS was mentioned by a few participants as important, as they would buy some food and drinks for a picnic at the POS. These shops were also important as they provided the opportunity to buy drinks after being active. POS should not be too close to a pub or bar because that would limit the possibilities to play, be active and make noise. *“I like it that there are many people here but I don’t want to bother them*, *they are eating and drinking at the restaurants and pubs and I don’t want to disturb them*.*”* (Girl 5, 15 years).

#### Features

**Fig 1:**
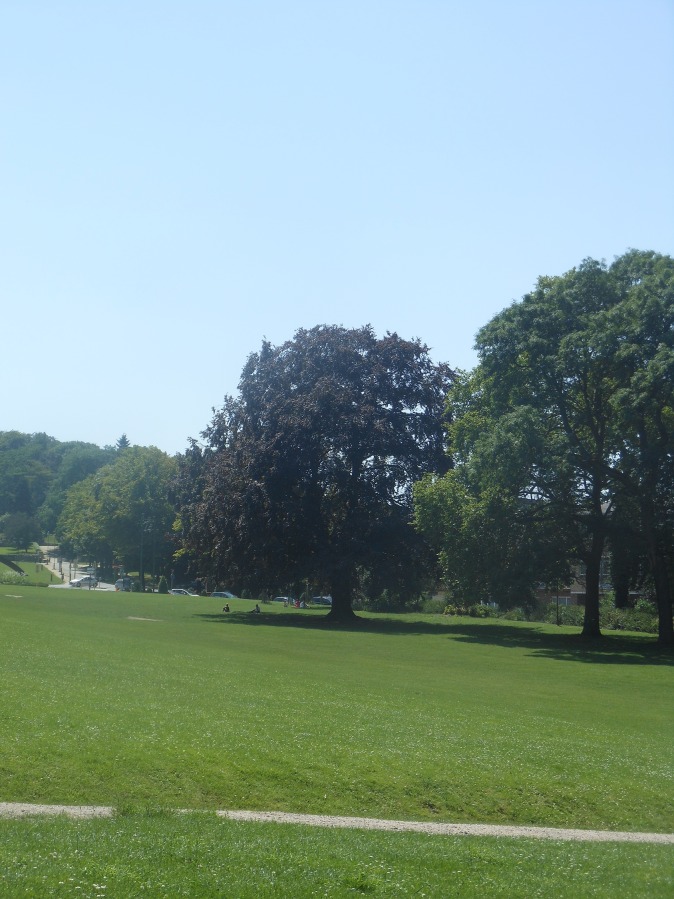
Large open spaces with grass and some trees encourage adolescents to be active. Park van Vorst, Brussels.

**Fig 2:**
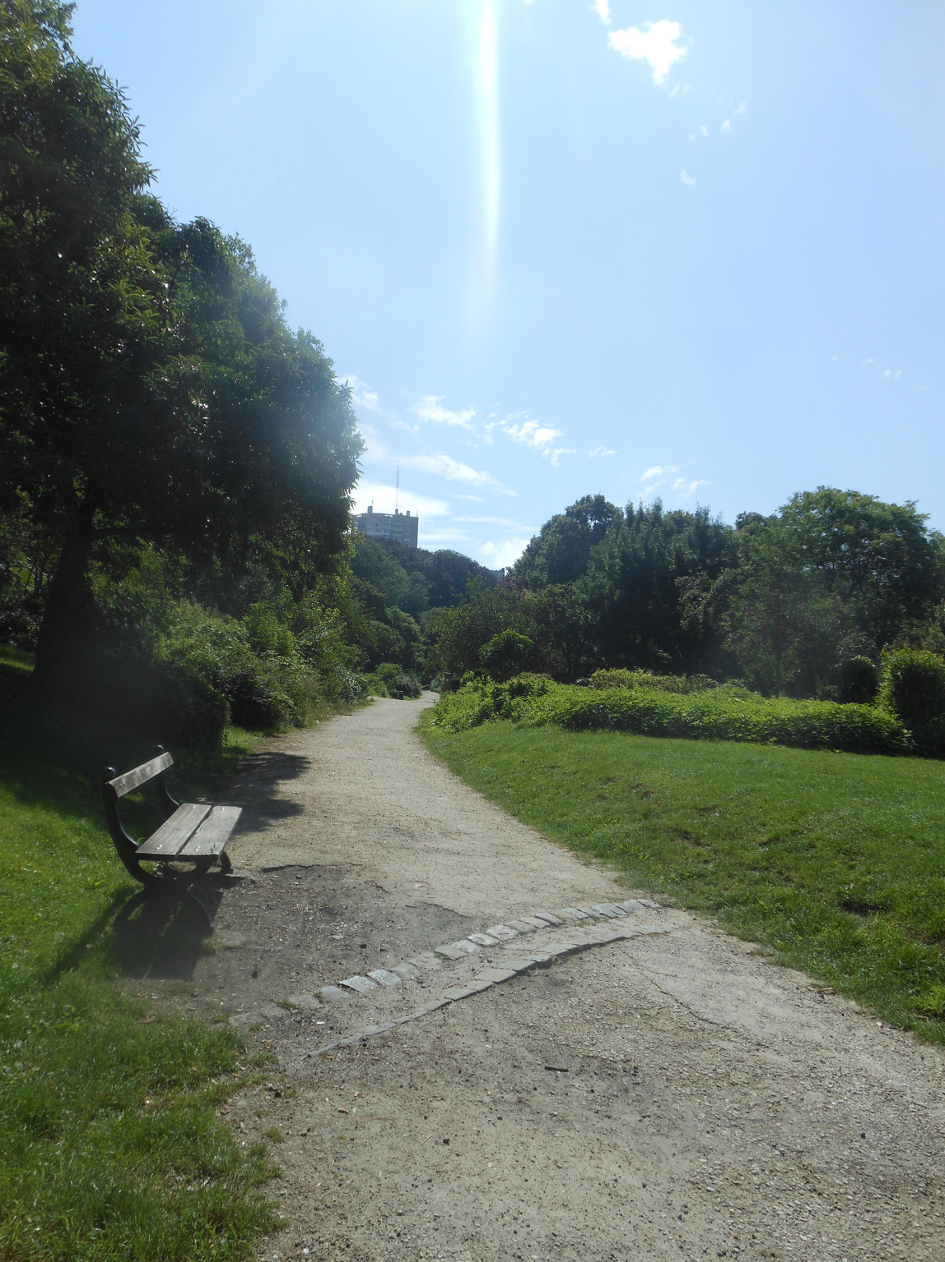
Paths with slopes are encouraging to be active for joggers and cyclists. This was mentioned by the participants because it increases the training intensity. Park van Vorst, Brussels.

Several kinds of features of POS were mentioned by the participants to attract them to visit a POS and to encourage them to be active. These features were of natural origin (e.g., ponds and water features, trees to climb, green space, slopes or paths) or man-made (e.g., play equipment, basketball rings, benches, BBQ spots, picnic shelters or toilets). Some participants mentioned large open spaces with some trees and grass as being important (Fig 1). “*What I like here are the trees and the grass to sit on*, *on a square you can’t sit on the ground or do anything and here we can have a picnic and do lots of other stuff*.*”* (Girl 4, 15 years). The size of the POS was also important for adolescents who like to play sports, such as soccer or basketball. *“This is a large space*, *and in some parks there are too many trees and not much space to play*. *It is not really supportive when there is a tree in the middle of the soccer field*.*”* (Boy 2, 12 years). For adolescents who like to jog or cycle in POS, paths with slopes were perceived as positive because they increase the training intensity (Fig 2).

**Fig 3:**
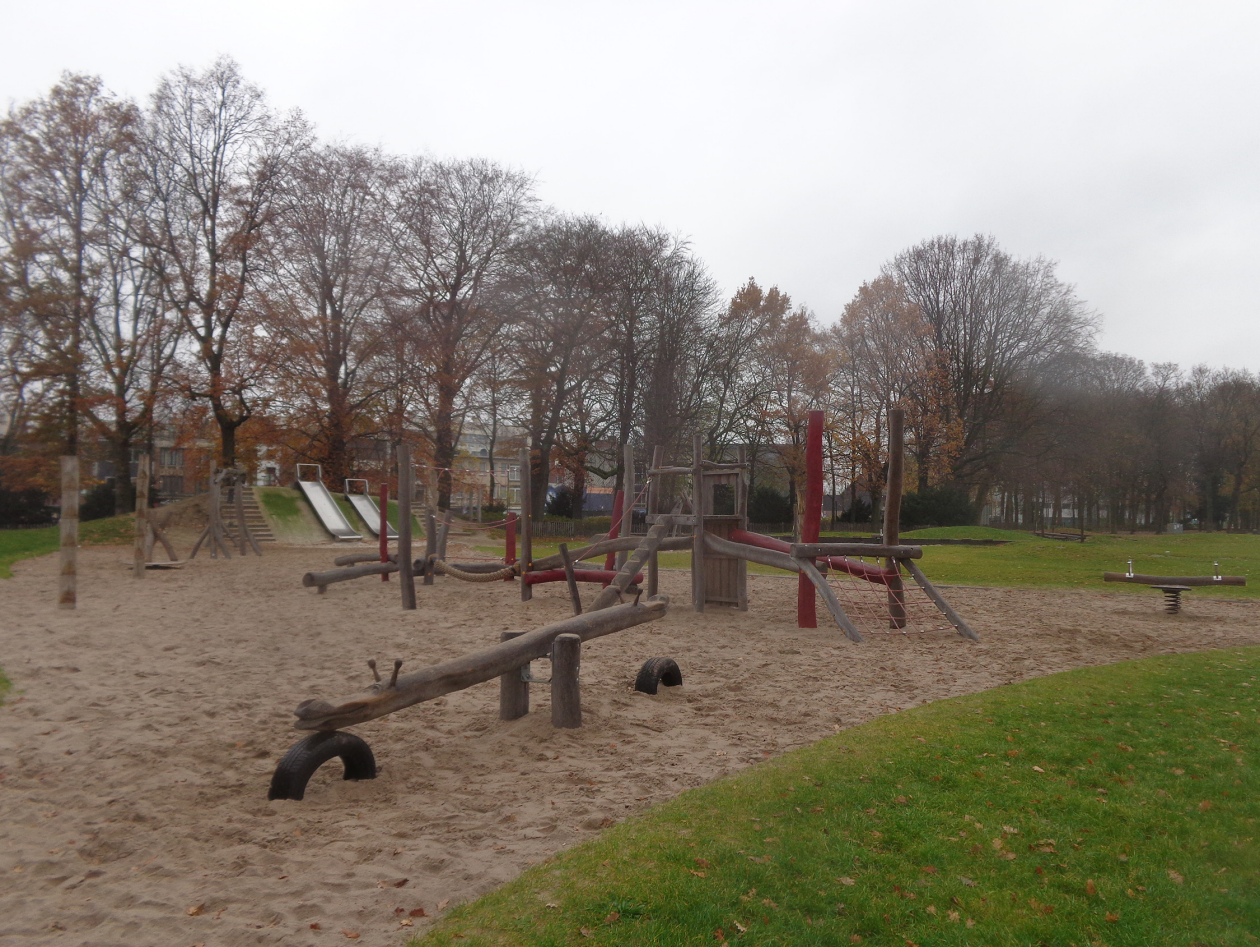
Playgrounds are important for younger adolescents (12–13 year olds) for POS visitation and physical activity. Sleepstraat Ghent.

**Fig 4:**
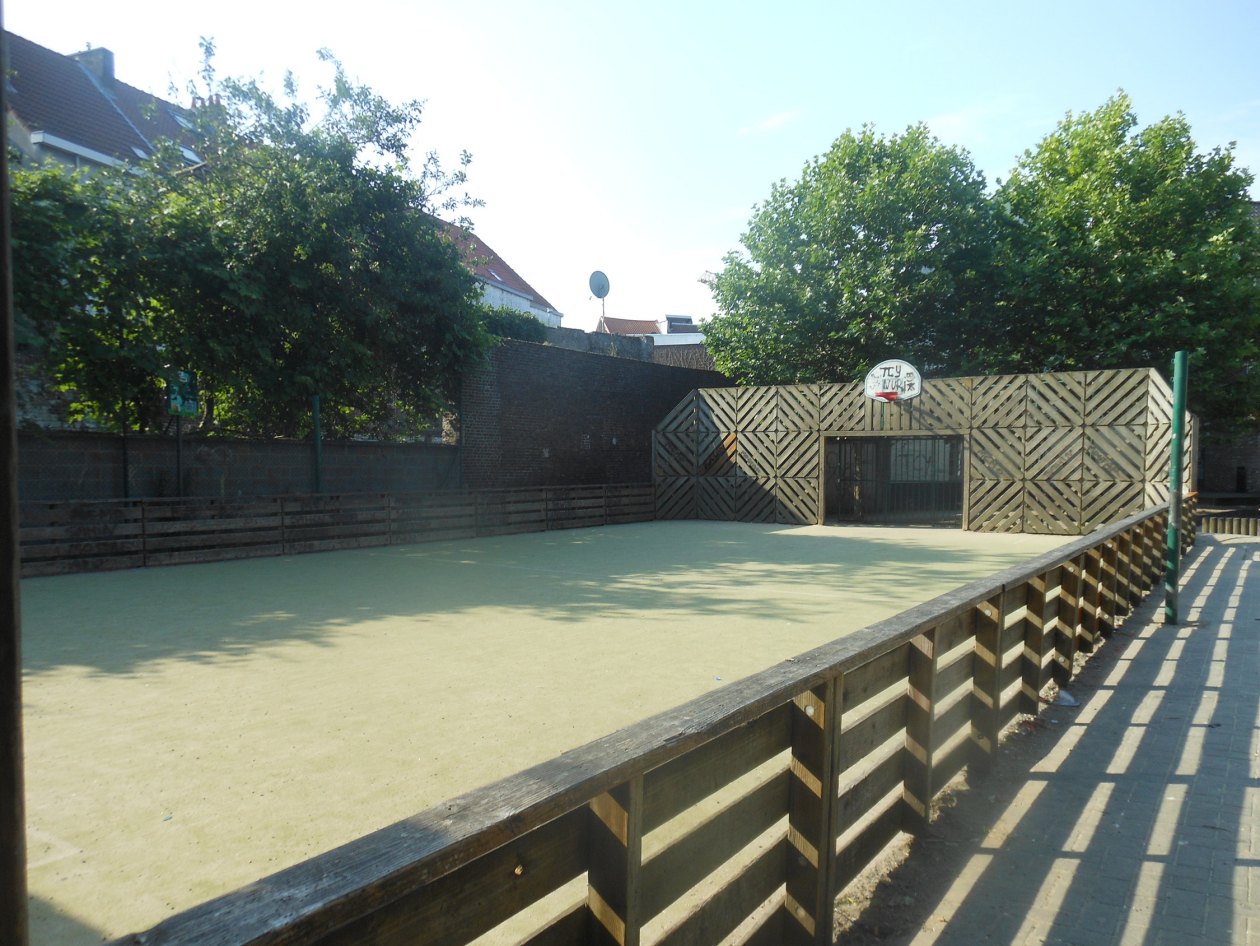
Sport fields were mostly pointed out as important by boys to visit and be active. Kielpark Antwerp.

Playgrounds and sport fields were considered attractive features for adolescents to visit and be active in POS (Figs 3 and 4). However, only the younger participants (12–13 years old) mentioned swings, slides, sandpits and wooden constructions as positive facilities where they can play hide and seek, tag or invent their own games. Many participants (mostly boys) mentioned sport fields and especially soccer fields as being important to encourage physical activity. However, sport facilities, such as badminton fields and table-tennis tables, were rarely used because participants did not have the necessary equipment to use the facilities. Other amenities such as water fountains, toilets, bicycle racks, sheds and tables encouraged adolescents to visit a POS.

Above all, the diversity of the features and thereby the possibility to engage in many different active and sedentary activities was important. Some participants also mentioned that they liked facilities that were appropriate for multiple age groups, so that adolescents who have to babysit their younger siblings can bring them to the POS.

#### Aesthetics

Many adolescents mentioned aesthetics of the POS to be important to visit a POS and to be active. The most important factor was the presence of nature and green. One 15-year-old girl said: “*I usually come here to jog*, *and when a place is green and open it motivates to be active*. *This is one of the reasons why I come to this park”*. (Girl 1, 15 years). Many participants indicated that these green settings encourage them to be active. A few adolescents also indicated that fresh air (produced by the trees in parks), singing of birds and insects they could examine, encouraged POS visitation.

**Fig 5:**
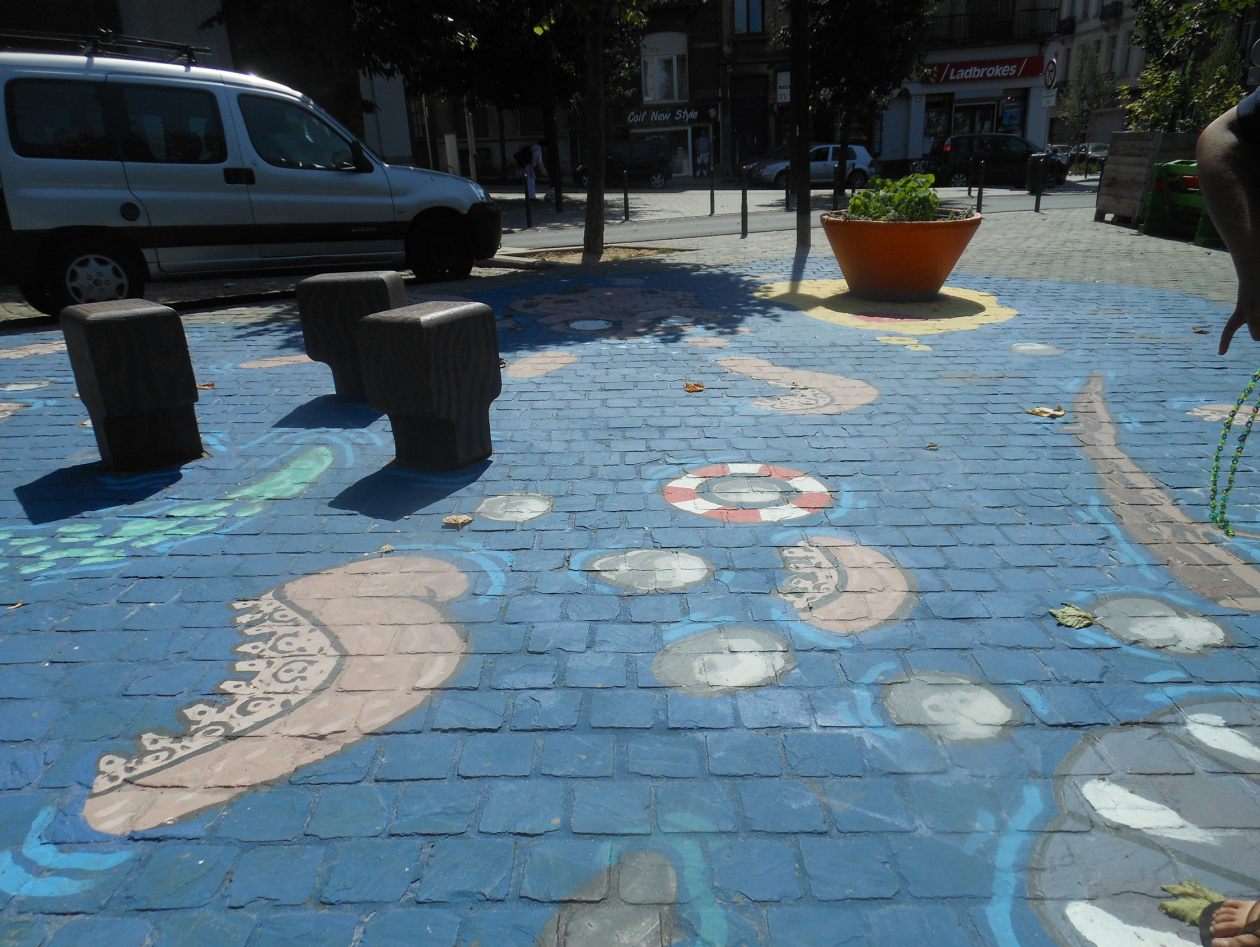
Ground paintings were perceived as attractive, unwanted graffiti as not attractive for visiting a POS. Bethlehemplein, Brussels and Rabotpark Ghent.

**Fig 6:**
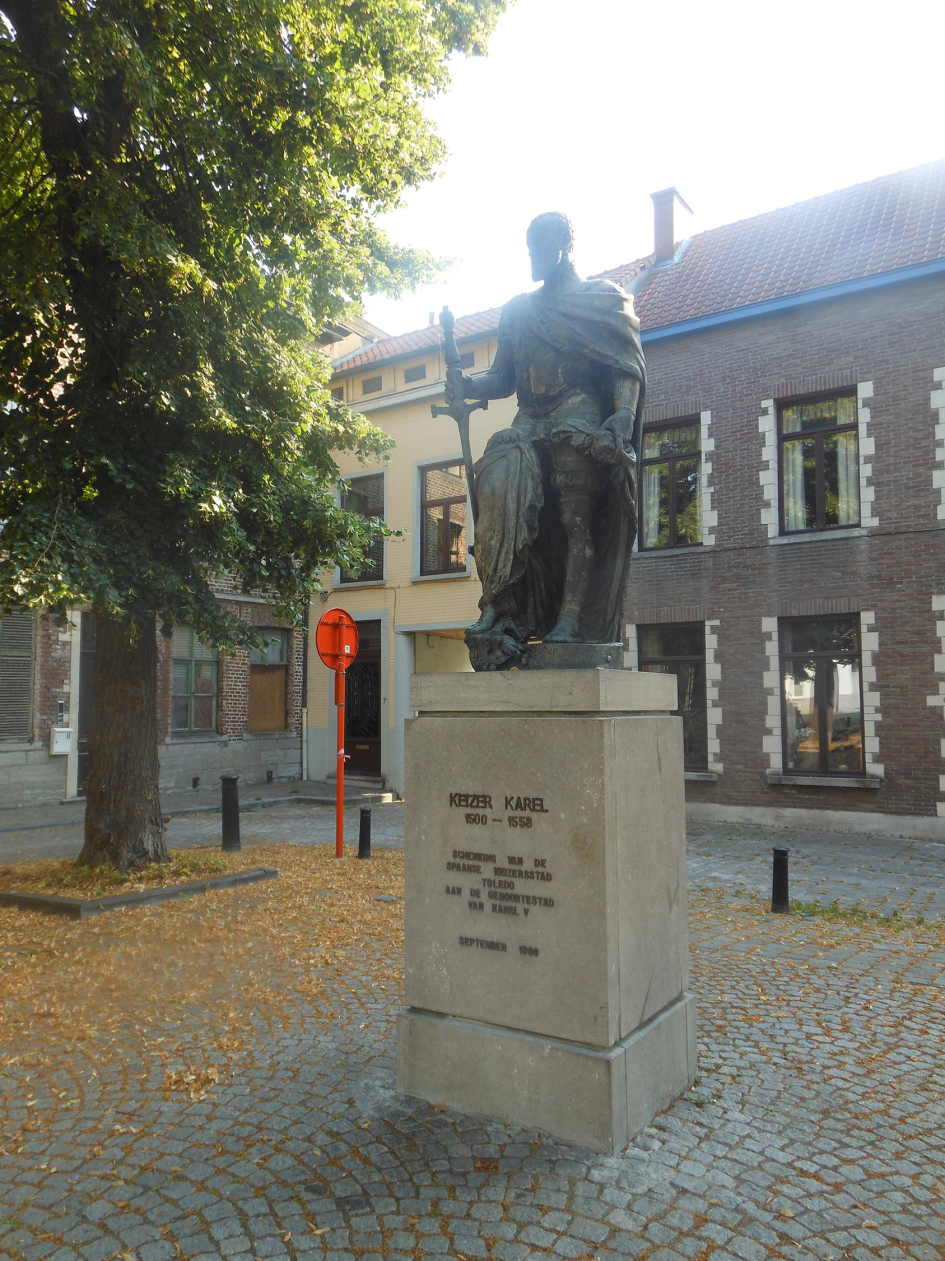
Historical elements and statues were perceived by some participants as attractive to visit a POS. Prinsenhofplein, Ghent.

It was indicated by a few participants that more colours in POS would be good. Graffiti was only accepted on a special graffiti wall or as a beautiful wall- or ground painting, not as “tags” (Fig 5). Few participants said they liked historical elements in POS as a landmark (e.g., to meet up at the big statue in the middle of the square). Furthermore, these historical elements (such as statues and old buildings) were liked because they illustrate the historical background of the city (Fig 6). A few adolescents said they liked to go to POS for rest and quietness, and other adolescents reported that loud music would discourage them from visiting the POS.

#### Upkeep

**Fig 7:**
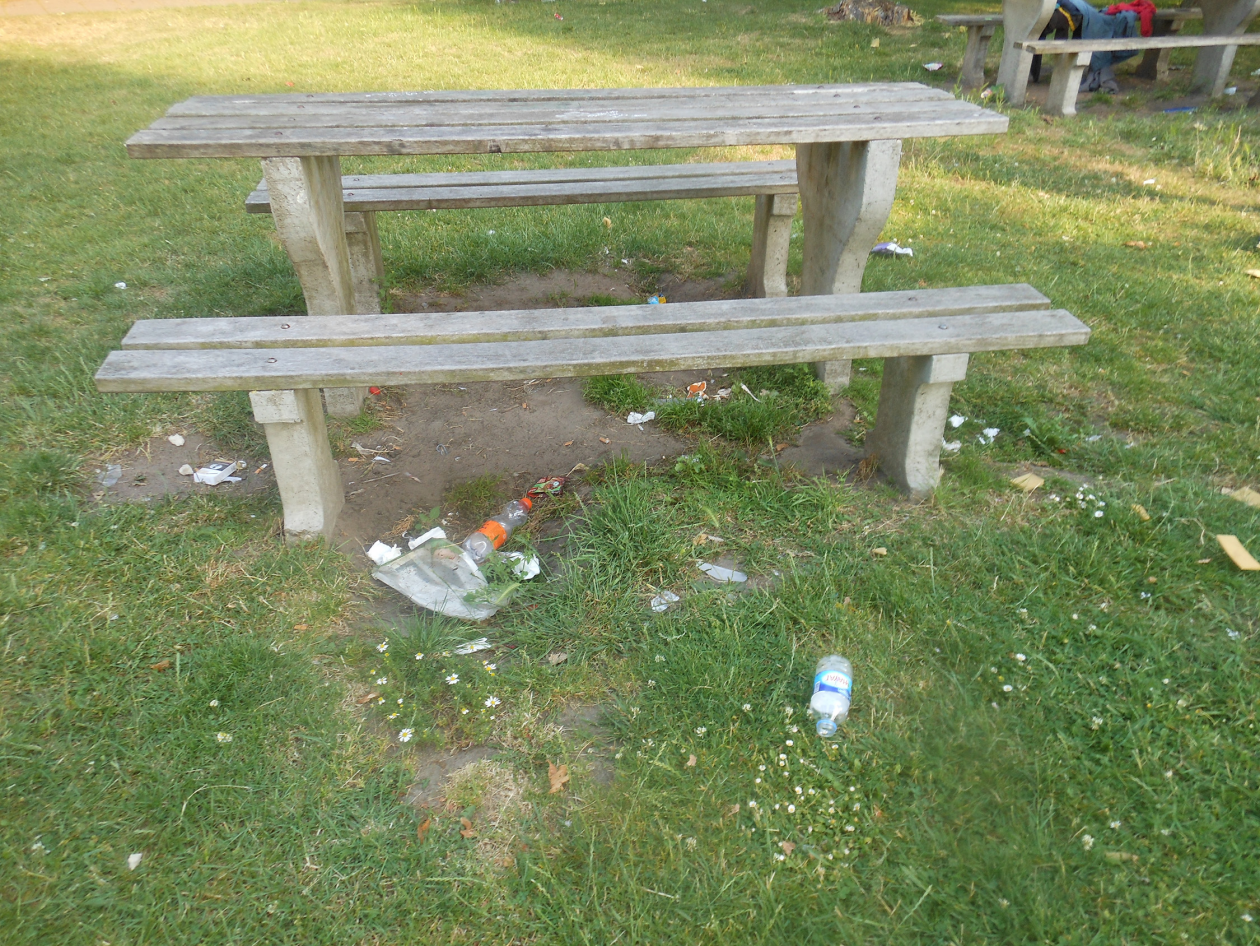
Garbage lying around was perceived to be unsupportive to visit a POS. Rabotpark, Ghent.

The cleanliness and upkeep of the POS were important for encouraging visitation and physical activity in the POS. Many participants mentioned garbage lying around (as a result of a lack of bins or full bins) (Fig 7), broken glass on the ground and illegal dumping of litter as factors that would discourage their visitation and physical activity. This is illustrated by a quote from a 14-year-old boy: “*The refuse collectors should come more often to clean the park*, *now there is garbage everywhere*. *We come here often for a picnic and there is only one bin around here and it is always full*. *And because it is always full everybody just throws garbage on the ground*. *The bin should be emptied more frequently and they should put a second bin somewhere over here*.*”* (Boy 10, 14 years).

The maintenance of the facilities and amenities was also mentioned by some participants as important for visiting the POS and being active. Maintenance of the surface of the playgrounds and sport fields was also mentioned by some participants. A playground or sport field encouraged physical activity when the surface was made of appropriate materials (e.g., no sand or concrete on a soccer field), there were no dangerous objects lying around (e.g., glass or stones) and when playing surfaces were even (no cracks). A 12-year-old boy mentioned: “*Although there are soccer goals here*, *we don’t play soccer here*. *There is sand here and it is not easy to play soccer on sand and there is garbage and glass on the soccer field and that is dangerous*. *Although there are no goals on the grass and it is located on a slope*, *we prefer to play on the grass since it is better maintained and there is no glass*.*”* (Boy 2, 12 years).

#### Physical aspects of safety

Physical aspects that negatively affected adolescents’ feelings of safety were insufficient lightning, poor maintenance of the facilities, too much traffic near the POS, and secluded areas or paths. Safe facilities for young children was also mentioned to be important by a few participants because many adolescents go to POS with their (younger) siblings. For example, one 12-year-old girl said: “*These stairs (with very high steps) here are too dangerous for young children*. *I think that it is important because I don’t like it when my younger siblings get hurt*.*”* (Girl 2, 12 years).

Secluded areas and paths were perceived unsafe for a few participants and were often avoided by adolescents. For example, a 13-year-old girl mentioned: *“I like it here because it is a large and open space and you can see everything that is going on*. *It is good if there are some trees but not too much otherwise it is too secluded*.*”* (Girl 6, 13 years). Similarly, a 14-year-old girl stated: *“[…] it is open here*, *if something would happen*, *there will always be someone who would see it and come and help*.*”* (Girl 3, 14 years). The absence of sufficient road crossings, traffic lights and cycle paths around the POS contributed to feeling unsafe in traffic and discouraged POS visitation: *“When I come out of the skate park*, *it is dangerous to cross the street here*, *it is a street with lots of traffic*. *There are some road crossings here but it would be better and safer if there were more*. *I think the cars would pay more attention*.*”* (Boy 9, 14 years).

#### Policy

Activities that were sporadically organized at the POS were perceived as positive for encouraging physical activity. These organized activities made it possible for adolescents to engage in a variety of activities such as badminton, tennis, and bouncy castle with equipment they do not have or cannot afford themselves and was perceived as a trigger to visit a POS and be active. A secluded area for dogs was perceived as positive by a few participants (i.e., non-dog-owners) whereas others preferred to let their dog walk around off-leash in the POS.

### Part 2: Social environment

#### Social network

When asked with whom participants visited the POS, 71% indicated that they usually visited it with friends, 46% with family, 14% said they usually visited the POS alone and an additional 3% with an organization such as scouts. Almost all participants attached great importance on the places where their friends and family (especially siblings) were or wanted to go. When asked why they would go to a specific POS, many participants answered: *“Because this is where my friends are*.*”* The importance of friends was illustrated by a quote from a 12-year-old boy: *“I find it really important that my friends are here*, *because every time I come here alone it is so boring and then I have to wait and wait till my friends come*.*”* (Boy 3, 12 years). A 15-year-old girl stated: *“I often run into people I know here*, *that really attracts me to come here*. *Depending on who I encounter*, *I may do something active or not*.*”* (Girl 5, 15 years).

Social contact in the POS was also mentioned by some participants to be important for visiting a POS and to be active. Almost all participants indicated that when they knew many people in the neighbourhood they were more likely to visit a POS as they felt safer. More youth in the POS to play with, was also perceived as positive by almost all participants. Therefore, POS not often used by adolescents (e.g., a small playground with features for small children, mostly used by mothers with young kids) were considered less attractive to visit and to be active.

#### Other users

The behaviour of other users (particularly adolescents older than 16 years) was something mentioned by almost all participants and it appeared to be a deterrent for visiting and being active in a POS. For example, *“I like to play soccer but sometimes when we are playing there are older kids who come here and they say we have to leave because it is their soccer field and that is not nice… or sometimes they take our ball and throw it away*. *So when there are older children here we go to another park with a soccer field so we can still play soccer*.*”* (Boy 5, 12 years).

Few adolescents mentioned that they would prefer not to go to places where there may be lots of people of a certain ethnicity, in order to avoid conflicts. *“One week ago there were some Bulgarian kids who wanted to play with us*, *but we said no and then it ended up in a fight*. *… We don’t like to play together because lots of them use drugs and smoke*.*”* (Boy 6, 13 years). However, another participant mentioned that the presence of people with different nationalities in a POS attracts her to go there. Some participants also mentioned that too many people in a POS would keep them from being physically active, implying that POS have to be large enough to provide enough space for everyone.

#### Social aspects of Safety

Safety is an issue that is linked to all other factors of the social environment and it was mentioned by all participants in some way. The feeling of safety was a very important factor for visiting a POS, but of less importance for the activity level of the participants. Feelings of safety were most affected by the presence of undesirable POS users. People “acting weird”, using drugs or drinking, involved in criminal affairs or harassing others (gangs) and homeless people discouraged almost all adolescents to visit or stay at a POS. *“I think it is a bit scary when there are people lying on the ground with booze*. *I don’t know why*, *it is just a feeling I have*. *[…] There are also sometimes people doing drugs here*. *This is why I would not come here in the evening and why I would make a detour when I am riding my bike instead of going through the park*.*”* (Girl 4, 15 years). Some participants mentioned a previous incident with an undesirable POS user that made them leave the POS and in some cases they indicated never using the POS again. “*We often met up here in the park with friends*, *but one time there were two men bothering us*. *We just took our stuff and went to my friend’s garden*. *Now we never go to that park again*.*”* (Girl 4, 15 years).

Participants seemed to be most affected by the undesirable users when there was actual interaction. *“There are often people doing drugs over there*, *but that has no influence on me*, *it doesn’t bother me*. *However*, *when there are gangs hanging around it does bother me to come here and to be active*. *Because they sometimes harass us*, *or most of the time*, *the girls*.*”* (Boy 19, 14 years). Feeling unsafe in the presence of undesirable users was mostly mentioned by girls. The presence of these undesirable users made the participants feel unsafe, however, for a few participants these feelings of insecurity were minimized when women with children or people they knew were in the POS.

The idea of being alone in a POS in the evening or at night was scary for some of the participants: *“I don’t think it is nice here in the evening*, *during the day there are many children and people walking around*, *but I think that in the evening it gets really quiet here and that doesn’t give me a safe feeling … When there are people and children around me I feel more at ease*, *it is good to know that there is someone to go to if I would have a problem … At night there are people sitting here on the benches and that is scary*.*”* (Girl 4, 15 years).

Some adolescents pointed out that when they trusted the other users in the POS, when people got along well and were willing to help, they felt safer, and therefore used the POS more often.

#### Parents

Parents still play an important role in adolescents’ decisions concerning POS visitation. A few of the participants said they go to one specific POS just because they were used to go there with their parents when they were younger. Some participants had to ask permission before going to a POS and were not allowed to go to a POS that was located too far away from home or where parents perceived it to be unsafe: *“We almost never go to the other place because we heard bad things happen there and there are often groups of men hanging around*, *sometimes taking drugs*. *That is why our parents do not allow us to go there*. *Only when it is totally empty we can go there together with our family*.*”* (Boy 7, 12 years).

#### Privacy

Privacy was an important issue for a few participants. Some of them especially went to POS to have some time alone, away from their parents. POS offer a good alternative (compared to sitting at home) for adolescents because at home it often gets crowded: *“At home we are always together and our house is not that big and it is always a bit crowded*. *When I come to this POS*, *there are more people here but it is less busy than at home*.*”* (Girl 5, 15 years). Few of the participants indicated that small secluded places are good when they want to be in a quiet place or alone with friends.

#### Modelling

Friends asking to hang out or do something together contributed to being active for almost all of the participants. The activity level of parents was less important. *“When someone starts playing with the ball*, *everybody gets active”* (Girl 6, 13 years) and *“My friends often ask to do something*, *today we went swimming*. *And when we go to the playground there are often other boys to play with and then we can choose whether we play together or not… So when I come here with friends we can ask others to join and when I am alone I can come here and make new friends*.*”* (Boy 3, 12 years). Active use of POS by others, was something that was perceived as supportive by many adolescents for the atmosphere and to be active. *“The park near my house is always empty and when I look out the window I want to see people playing*, *running*, *moving … and then I would want to go out immediately and play along*.*”* (Girl 10, 14 years).

## Discussion

The objective of this study was to investigate the physical and social characteristics of POS in low SES neighbourhoods that affect adolescents’ POS visitation and physical activity in POS. Walk-along interviews were used to obtain context-specific and detailed information. The results show that there are different aspects of the physical and social environment that affect visitation and physical activity in a POS. Moreover, there was a substantial overlap in factors that affect visitation and physical activity in POS. Adolescents’ (active) use of POS was affected by the following aspects of the physical environment: accessibility and location, features, aesthetics, upkeep, safety and policy. The aspects of the social environment that affected adolescents’ (active) use included the social network, presence of other users, safety, parents, privacy and modelling.

During the analysis of the interviews it became clear that it is almost impossible to completely separate the physical and social environmental factors that affect (active) use of POS. This is supported by the ecological model of health behaviour, stating that several levels influence physical activity [15], and that interactions exist between the different levels. For example, some participants indicated that they thought it was important to have facilities for the young and old (= physical environment) so that they can visit a POS with their siblings, parents and grandparents (= social environment). The interplay between these environments may exist in two directions: strengthen a positive or negative impact or counteract the impact. For example, the impact of a positive physical environment (e.g., a POS with well-maintained and age-appropriate features) can be reinforced with a positive social environment (e.g., lots of other adolescents to play with), or the impact of a positive physical environment can be counteracted when the social environment is unsupportive (e.g., adolescents feel unsafe because of the presence of undesirable users). Whether physical or social environmental characteristics dominate the decision to visit or to be active in a POS appeared to be strongly dependent on personal experiences, beliefs and the specific purpose of visiting a POS. This indicates that researchers should consider both physical and social factors of POS to generate a more comprehensive view on the characteristics that influence whether or not adolescents visit and use a POS for physical activity.

Our results indicate that the social environment of POS may play a more important role than the physical environment. The presence of other adolescents and friends encouraged POS visitation and physical activity. This was also found in previous studies with younger adolescents in the US (9–13 years and 12–14 years old [65, 66]). Consistent with previous literature, the presence of other adolescents and friends and family who are physically active in a POS encouraged adolescents to be active in POS themselves [40, 67]. The presence of others (whether or not active) appeared more important than physical characteristics. This indicates that POS with a positive social and physical environment should be created and that exclusively investing in the physical attributes of POS may not be sufficient. Only a few participants mentioned organized activities in POS, possibly indicating that there are not many activities organized in the POS where the interviews took place. However, organized activities offer a good opportunity to create a supportive social environment and to encourage low SES youth to be active.

Our findings showed that accessibility (POS located close to home and easy accessible by foot, bike and public transport) was important for choosing a POS to go to, due to limited independent mobility of adolescents compared to adults. However, almost all participants mentioned that specific features (e.g., sport facilities) and social factors (e.g., friends) could encourage them to visit a POS located further away. This finding could possibly explain the inconsistencies found in previous literature concerning the relationship between accessibility of POS and adolescents’ physical activity [25, 28–32]. Previous research has investigated the POS closest to the adolescents’ home assuming that these are the most frequently used POS. However, this study showed that POS located further, with a positive physical and social environment may also be used. Although accessibility was not mentioned to affect physical activity directly, a POS that is easily accessible by foot or bike can contribute to overall physical activity. Our results revealed that many adolescents used active transportation to travel to a POS. This is consistent with previous research which showed that active transportation was used most frequently among youth to travel to a park [17, 68]. Moreover, adolescents’ active transportation is positively associated with daily physical activity [37]. Creating easy accessible and attractive POS can lead to higher amounts of active transportation to POS among youth and thereby increase overall physical activity. Thus, total physical activity levels can be increased, even when adolescents visit a POS to engage in sitting activities.

The most frequently mentioned modifiable physical factors for visiting and active use of a POS for both boys and girls were natural features, playgrounds (mostly younger participants) and sport fields. However, the use of these sport fields and playgrounds may depend on the type of sport and play facilities that are present, as some participants did not have the right equipment to use these facilities (e.g., badminton racquets). Some physical attributes that affected feelings of safety were linked to fear of getting hurt (e.g., poor maintenance of the facilities). This was mostly mentioned by girls which is consistent with previous literature [38, 69]. In our study, participants indicated road safety (e.g., insufficient pedestrian crossings) was important for POS visitation. In the review of Carver et al., no association was found between road safety and physical activity in adolescents [70]. Fear from crime (for example caused by secluded paths) was also mentioned, comparable with the results of the qualitative study conducted by Ries et al. (2008) [38].

The social aspects of safety that affected adolescents visiting and being active in a POS were undesirable users, number of other users and safety at night. The review of Carver et al. (2008), on the influence of neighbourhood safety on physical activity in children (7–16 years), revealed no association between stranger danger and physical activity [70]. This is in line with our finding that undesirable users discouraged visitation, but were not mentioned to affect physical activity in POS. Additionally, when mothers with children were present in a POS the negative impact of the undesirable users could be counteracted, which indicates that a POS with facilities for all ages can provide an environment where adolescents feel more safe.

### Practical Implications

For youth living in low SES neighbourhoods, POS can provide opportunities to be active outdoors. Urban planners and governments should try to create neighbourhoods with a positive social and physical environment. Possible ways to create a supportive social environment are: organizing events and activities in POS, providing sufficient lighting and no secluded places to increase social control. POS should be easily accessible and facilities that require (expensive) additional equipment (such as badminton racquets) should not be installed unless appropriate equipment is provided. Features for all ages are important for adolescents, such that they can visit a POS with friends and family. When designing a POS, both natural elements and sports- and play features appropriate for multiple age groups should be included. A variety of features that provide opportunities for different types of physical and social activities together with additional amenities (such as toilets) should be incorporated in new POS. Additionally, we found that adolescents also attach importance to POS with lots of colours (e.g., wall/ground paintings) and landmarks (e.g., a statue). When renovating existing POS, monuments and colours can easily be added. Furthermore, more attention should be paid by the city government to the maintenance of the features and surfaces of sport fields as this was a key factor for physical activity in POS (mainly for boys).

### Strengths and Limitations

The current study has some limitations that should be taken into account. First, this study tried to include all kinds of POS like vacant lots, parks, squares, playgrounds, streets and parking lots. However, interviews were only conducted in parks, skate parks, squares and sport fields/playgrounds because no adolescents were found in other locations (vacant lots, parking lots, streets). This possibly indicates that parks, skate parks, sport fields/playgrounds and squares are the POS that are used most frequently by adolescents in Belgian cities. In addition, the results were not analysed separately for each type of POS, therefore, it was not possible to determine if differences were observed for these different types of POS. Another limitation is that this research only focused on urban POS, and future research should include both urban and rural settings to obtain a more complete overview. In addition, the interviews were only conducted in neutral to good weather (i.e., not raining), and other characteristics of POS may be important when the weather is poor (i.e., in winter months). Further, due to the qualitative character of the research, it was not possible to define which aspect of the POS was most decisive for adolescents to visit POS or for physical activity in POS.

Most previous studies have investigated the attributes of the POS closest to the adolescents’ home assuming that these are the most frequently used POS. However, adolescents may visit other places and, therefore, it is important to investigate the environment that is actually used by the adolescents. This problem is known as the Modifiable Areal Unit Problem (MAUP) [71]. A strength of this study is that participants were interviewed in POS they frequently visited as well as POS they did not use. This way MAUP was avoided. Another strength of this study is the methodology used. When indoor sitting interviews are used, it is often difficult for the participants to recall specific details or perceptions of the environment. The walk-along interviews provided in-depth and context-specific information of a POS and observations of the participant whilst in the POS. This paper provides a detailed picture of all characteristics important for adolescents living in low SES neighbourhoods for visiting and being active in POS. Additionally, we examined the interplay between the physical and social environment. Future research on the physical environment should take the social environment and the interactions between the physical and social environment into account.

### Conclusion

This research revealed that both physical and social characteristics of POS may affect adolescents’ POS visitation and physical activity. Moreover, it is the combination of multiple factors that affect adolescents’ behaviour in POS and social factors are often more influential. Therefore, it is important for designers, urban planners and researchers to focus on physical as well as social characteristics of POS. Our findings emphasize that the combination of multiple physical as well as social environmental characteristics of POS will define the attractiveness of a POS to visit and to be active. This qualitative study is a good basis for further quantitative research to examine the most important characteristics of POS, in order to create a supportive environment for adolescents to be physically active.

## References

[1] Janssen I, Leblanc AG. Systematic review of the health benefits of physical activity and fitness in school-aged children and youth. Int J Behav Nutr Phys Act. 2010;7:40 10.1186/1479-5868-7-40 20459784 PMC2885312

[2] Hallal PC, Victora CG, Azevedo MR, Wells JC. Adolescent physical activity and health: a systematic review. Sports Med. 2006;36(12):1019–30. 17123326 10.2165/00007256-200636120-00003

[3] Dumith SC, Gigante DP, Domingues MR, Kohl HW III. Physical activity change during adolescence: a systematic review and a pooled analysis. Int J Epidemiol. 2011;40(3):685–98. 10.1093/ije/dyq272 21245072

[4] Riddoch CJ, Andersen LB, Wedderkopp N, Harro M, Klasson-Heggebo L, Sardinha LB, et al Physical activity levels and patterns of 9-and 15-yr-old European children. Med Sci Sports Exerc. 2004;36(1):86–92. 10.1249/01.Mss.0000106174.43932.92 14707773

[5] Ortega FB, Konstabel K, Pasquali E, Ruiz JR, Hurtig-Wennlof A, Maestu J, et al Objectively measured physical activity and sedentary time during childhood, adolescence and young adulthood: a cohort study. PLoS One. 2013;8(4):e60871 10.1371/journal.pone.0060871 23637772 PMC3634054

[6] Scheerder J, Vandermeerschen H, Bogers J, Thibaut E, Vos S. Vlaanderen Sport! Vier decennia sportbeleid en sportparticipatie Gent: Academia Press; 2013.

[7] Tafforeau J. Gezondheidsenquête. Belgium: Wetenschappelijk instituut Volksgezondheid; 2008 2008.

[8] Gordon-Larsen P, McMurray RG, Popkin BM. Determinants of adolescent physical activity and inactivity patterns. Pediatrics. 2000;105(6):E83 10835096 10.1542/peds.105.6.e83

[9] Vandendriessche JB, Vandorpe BF, Vaeyens R, Malina RM, Lefevre J, Lenoir M, et al Variation in sport participation, fitness and motor coordination with socioeconomic status among Flemish children. Pediatr Exerc Sci. 2012;24(1):113–28. 22433257 10.1123/pes.24.1.113

[10] Hanson MD, Chen E. Socioeconomic status and health behaviors in adolescence: a review of the literature. Journal of Behavioral Medicine. 2007;30(3):263–85. 10.1007/s10865-007-9098-3 17514418

[11] Moore LV, Diez Roux AV, Evenson KR, McGinn AP, Brines SJ. Availability of recreational resources in minority and low socioeconomic status areas. Am J Prev Med. 2008;34(1):16–22. 10.1016/j.amepre.2007.09.021 18083446 PMC2254179

[12] Eime RM, Harvey JT, Craike MJ, Symons CM, Payne WR. Family support and ease of access link socio-economic status and sports club membership in adolescent girls: a mediation study. Int J Behav Nutr Phys Act. 2013;10:50 10.1186/1479-5868-10-50 23618407 PMC3639833

[13] Humbert ML, Chad KE, Spink KS, Muhajarine N, Anderson KD, Bruner MW, et al Factors that influence physical activity participation among high- and low-SES youth. Qualitative Health Research. 2006;16(4):467–83. 10.1177/1049732305286051 16513991

[14] Romero AJ. Low-income neighborhood barriers and resources for adolescents' physical activity. Journal of Adolescent Health. 2005;36(3):253–9. 10.1016/j.jadohealth.2004.02.027 15737782

[15] Sallis JF, Cervero RB, Ascher W, Henderson KA, Kraft MK, Kerr J. An ecological approach to creating active living communities. Annual Review of Public Health. 2006;27:297–322. 10.1146/annurev.publhealth.27.021405.102100 16533119

[16] Carver A, Timperio A, Hesketh K, Crawford D. Are children and adolescents less active if parents restrict their physical activity and active transport due to perceived risk? Soc Sci Med. 2010;70(11):1799–805. 10.1016/j.socscimed.2010.02.010 20347200

[17] Veitch J, Carver A, Hume C, Crawford D, Timperio A, Ball K, et al Are independent mobility and territorial range associated with park visitation among youth? Int J Behav Nutr Phys Act. 2014;11:73 10.1186/1479-5868-11-73 24909862 PMC4061522

[18] Dunton GF, Whalen CK, Jamner LD, Floro JN. Mapping the social and physical contexts of physical activity across adolescence using ecological momentary assessment. Ann Behav Med. 2007;34(2):144–53. 10.1007/bf02872669 17927553

[19] Duzenli T, Bayramoglu E, Ozbilen A. Needs and preferences of adolescents in open urban spaces. Scientific Research and Essays. 2010;5(2):201–16.

[20] Timperio A, Giles-Corti B, Crawford D, Andrianopoulos N, Ball K, Salmon J, et al Features of public open spaces and physical activity among children: findings from the CLAN study. Prev Med. 2008;47(5):514–8. 10.1016/j.ypmed.2008.07.015 18718847

[21] Madanipour A. Whose public space?: international case studies in urban design and development Abingdon, Oxon; New York: Routledge; 2010.

[22] Light A, Smith JM. Philosophy and geography II: the production of public space Philosophy and geography,. Lanham: Rowman & Littlefield Publishers; 1998.

[23] Carmona M, Tiesdell S, Heath T, Oc T. Public places \2015 urban spaces: the dimensions of urban design 2nd ed. ed. Oxford: Architectural Press; 2010.

[24] Carr S. Public space Cambridge University Press; 1992.

[25] Ding D, Sallis JF, Kerr J, Lee S, Rosenberg DE. Neighborhood Environment and Physical Activity Among Youth A Review. Am J Prev Med. 2011;41(4):442–55. 10.1016/j.amepre.2011.06.036 21961474

[26] de Vet E, de Ridder DTD, de Wit JBF. Environmental correlates of physical activity and dietary behaviours among young people: a systematic review of reviews. Obes Rev. 2011;12(501):e130–e42. 10.1111/j.1467-789X.2010.00784.x 20630024

[27] Safron M, Cislak A, Gaspar T, Luszczynska A. Micro-environmental characteristics related to body weight, diet, and physical activity of children and adolescents: a systematic umbrella review. Int J Environ Health Res. 2011;21(5):317–30. 10.1080/09603123.2011.552713 21547807

[28] Ferreira I, van der Horst K, Wendel-Vos W, Kremers S, van Lenthe FJ, Brug J. Environmental correlates of physical activity in youth—a review and update. Obes Rev. 2007;8(2):129–54. OBR264 [pii] 10.1111/j.1467-789X.2006.00264.x 17300279

[29] Babey SH, Wolstein J, Krumholz S, Robertson B, Diamant AL. Physical activity, park access, and park use among California adolescents. Policy Brief UCLA Cent Health Policy Res. 2013(PB2013-2):1–8. 23599982

[30] Norman GJ, Nutter SK, Ryan S, Sallis JF, Calfas KJ, Patrick K. Community Design and Access to Recreational Facilities as Correlates of Adolescent Physical Activity and Body-Mass Index. J Phys Act Health. 2006;3:S118–S28. 28834510 10.1123/jpah.3.s1.s118

[31] Sallis JF, Prochaska JJ, Taylor WC. A review of correlates of physical activity of children and adolescents. Med Sci Sports Exerc. 2000;32(5):963–75. 10795788 10.1097/00005768-200005000-00014

[32] Van Der Horst K, Paw MJ, Twisk JW, Van Mechelen W. A brief review on correlates of physical activity and sedentariness in youth. Med Sci Sports Exerc. 2007;39(8):1241–50. 10.1249/mss.0b013e318059bf35 17762356

[33] Serrano-Sanchez JA, Lera-Navarro A, Dorado-Garcia C, Gonzalez-Henriquez JJ, Sanchis-Moysi J. Contribution of individual and environmental factors to physical activity level among Spanish adults. PLoS One. 2012;7(6):e38693 10.1371/journal.pone.0038693 22685598 PMC3369927

[34] Lloyd K, Burden J, Kiewa J. Young Girls and Urban Parks: Planning for Transition Through Adolescence. Journal of Park & Recreation Administration. 2008;26(3):21–38.

[35] Ries AV, Voorhees CC, Roche KM, Gittelsohn J, Yan AF, Astone NM. A Quantitative Examination of Park Characteristics Related to Park Use and Physical Activity Among Urban Youth. Journal of Adolescent Health. 2009;45(3):S64–S70. 10.1016/j.jadohealth.2009.04.020 19699439

[36] Janssen I, Rosu A. Undeveloped green space and free-time physical activity in 11 to 13-year-old children. Int J Behav Nutr Phys Act. 2015;12(1):26 10.1186/s12966-015-0187-3 25886212 PMC4340861

[37] Chillon P, Ortega FB, Ruiz JR, De Bourdeaudhuij I, Martinez-Gomez D, Vicente-Rodriguez G, et al Active commuting and physical activity in adolescents from Europe: results from the HELENA study. Pediatr Exerc Sci. 2011;23(2):207–17. 21633133 10.1123/pes.23.2.207

[38] Ries AV, Gittelsohn J, Voorhees CC, Roche KM, Clifton KJ, Astone NM. The environment and urban adolescents' use of recreational facilities for physical activity: A qualitative study. American Journal of Health Promotion. 2008;23(1):43–50. 10.4278/ajhp.07043042 18785374

[39] Ries AV, Voorhees CC, Gittelsohn J, Roche KM, Astone NM. Adolescents' perceptions of environmental influences on physical activity. Am J Health Behav. 2008;32(1):26–39. 10.5555/ajhb.2008.32.1.26 18021031

[40] de Farias JC Junior, Lopes Ada S, Mota J, Santos MP, Ribeiro JC, Hallal PC. Perception of the social and built environment and physical activity among Northeastern Brazil adolescents. Prev Med. 2011;52(2):114–9. 10.1016/j.ypmed.2010.12.002 21147155

[41] Baran PK, Smith WR, Moore RC, Floyd MF, Bocarro JN, Cosco NG, et al Park use among youth and adults: examination of individual, social and urban form factors. Environment and Behavior. 2014;46(6):768–800.

[42] Floyd MF, Bocarro JN, Smith WR, Baran PK, Moore RC, Cosco NG, et al Park-based physical activity among children and adolescents. Am J Prev Med. 2011;41(3):258–65. 10.1016/j.amepre.2011.04.013 S0749-3797(11)00327-8 [pii]. 21855739

[43] Kaczynski AT, Potwarka LR, Saelens BE. Association of park size, distance, and features with physical activity in neighborhood parks. Am J Public Health. 2008;98(8):1451–6. 10.2105/ajph.2007.129064 18556600 PMC2446450

[44] Carpiano RM. Come take a walk with me: the "go-along" interview as a novel method for studying the implications of place for health and well-being. Health Place. 2009;15(1):263–72. 10.1016/j.healthplace.2008.05.003 18606557

[45] Loebach JGJ. Child-led tours to uncover childrens' perceptions and use of neighborhoud environments. Children, Youth and environments. 2010;20(1):53–90.

[46] Estabrooks PA, Lee RE, Gyurcsik NC. Resources for physical activity participation: Does availability and accessibility differ by neighborhood socioeconomic status? Ann Behav Med. 2003;25(2):100–4. 10.1207/S15324796abm2502_05 12704011

[47] Stad Antwerpen in Cijfers. 2015;3:25. Available: http://www.antwerpen.buurtmonitor.be/

[48] De Wijkmonitoring van het Brussels Hoofdstedelijk Gewest. 2015;3:25. Available: https://wijkmonitoring.irisnet.be/maps/

[49] Gent in cijfers.2015;3:25. Available: http://gent.buurtmonitor.be/

[50] Statistics Belgium. 2015;3:25. Available: http://statbel.fgov.be/nl/modules/publications/statistiques/arbeidsmarkt_levensomstandigheden/Fiscale_inkomens_-_per_gemeente_-_2008.jsp

[51] Shaw C, Louca-Mai B, Davey C. Guidelines for research with children and young people: NCB Research Centre 2011.

[52] Ruiz-Canela M L-dBC, Carlos S, Calatrava M, Beltramo C, Osorio A, et al Observational research with adolescents: a framework for the management of the parental permission. BMC Med Ethics [Research Support, Non-US Gov't]. 2013;14(2). 10.1186/1472-6939-14-2 PMC3585740 23286743

[53] Belgian Official Journal. The privacy act. 2015;12:8. 1993. Available: https://www.privacycommission.be/en/privacy-act.

[54] Lien N, Friestad C, Klepp KI. Adolescents' proxy reports of parents socioeconomic status: How alid are they? Journal of Epidemiological community health. 2001;55:731–7. 10.1136/jech.55.10.731 PMC1731778 11553657

[55] Veitch J, Salmon J, Carver A, Timperio A, Crawford D, Fletcher E, et al A natural experiment to examine the impact of park renewal on park-use and park-based physical activity in a disadvantaged neighbourhood: the REVAMP study methods. BMC Public Health. 2014;14:600 10.1186/1471-2458-14-600 24924919 PMC4073813

[56] Mujahid MS, Diez Roux AV, Morenoff JD, Raghunathan T. Assessing the measurement properties of neighborhood scales: From psychometrics to ecometrics. Am J Epidemiol. 2007;165(8):858–67. 17329713 10.1093/aje/kwm040

[57] McNeill LH, Kreuter MW, Subramanian SV. Social environment and physical activity: a review of concepts and evidence. Soc Sci Med. 2006;63(4):1011–22. 10.1016/j.socscimed.2006.03.012 16650513

[58] Sampson RJ, Raudenbush SW, Earls F. Neighborhoods and violent crime: a multilevel study of collective efficacy. Science. 1997;277(5328):918–24. 9252316 10.1126/science.277.5328.918

[59] Charmaz K. Constructing grounded theory London: Sage; 2014.

[60] McCormack GR, Rock M, Toohey AM, Hignell D. Characteristics of urban parks associated with park use and physical activity: a review of qualitative research. Health Place. 2010;16(4):712–26. 10.1016/j.healthplace.2010.03.003 20356780

[61] Bedimo-Rung AL, Mowen AJ, Cohen DA. The significance of parks to physical activity and public health: a conceptual model. Am J Prev Med. 2005;28(2 Suppl 2):159–68. 10.1016/j.amepre.2004.10.024 15694524

[62] Sandelowski M. Real qualitative researchers do not count: the use of numbers in qualitative research. Research in nursing & health. 2001;24(3):230–40. 11526621 10.1002/nur.1025

[63] Van Cauwenberg J, Van Holle V, Simons D, Deridder R, Clarys P, Goubert L, et al Environmental factors influencing older adults' walking for transportation: a study using walk-along interviews. Int J Behav Nutr Phys Act. 2012;9:85 10.1186/1479-5868-9-85 22780948 PMC3499291

[64] Lenders S. Afbakening van het Vlaamse platteland—een statistische analyse -.2015;4:1. 2005. Available: http://www2.vlaanderen.be/landbouw/downloads/volt/38.pdf

[65] Dunton GF, Kawabata K, Intille S, Wolch J, Pentz MA. Assessing the Social and Physical Contexts of Children's Leisure-Time Physical Activity: An Ecological Momentary Assessment Study. American Journal of Health Promotion. 2012;26(3):135–42. 10.4278/ajhp.100211-QUAN-43 22208410

[66] Smith AL, Troped PJ, McDonough MH, DeFreese JD. Youth perceptions of how neighborhood physical environment and peers affect physical activity: a focus group study. Int J Behav Nutr Phys Act. 2015;12:80 10.1186/s12966-015-0246-9 26091859 PMC4487508

[67] Santos MP, Page AS, Cooper AR, Ribeiro JC, Mota J. Perceptions of the built environment in relation to physical activity in Portuguese adolescents. Health Place. 2009;15(2):548–52. 10.1016/j.healthplace.2008.08.006 19004663

[68] Smith L, Sahlqvist S, Ogilvie D, Jones A, Griffin SJ, van Sluijs E. Is active travel to non-school destinations associated with physical activity in primary school children? Prev Med. 2012;54(3–4):224–8. 10.1016/j.ypmed.2012.01.006 22285945 PMC3856476

[69] Edwards N, Hooper P, Knuiman M, Foster S, Giles-Corti B. Associations between park features and adolescent park use for physical activity. Int J Behav Nutr Phys Act. 2015;12(1):21 10.1186/s12966-015-0178-4 25879200 PMC4341879

[70] Carver A, Timperio A, Crawford D. Playing it safe: the influence of neighbourhood safety on children's physical activity. A review. Health Place. 2008;14(2):217–27. 10.1016/j.healthplace.2007.06.004 17662638

[71] Kwan MP. The Uncertain Geographic Context Problem. Annals of the Association of American Geographers. 2012;102(5):958–68. 10.1080/00045608.2012.687349

